# Construction of a bacterial surface display system based on outer membrane protein F

**DOI:** 10.1186/s12934-019-1120-2

**Published:** 2019-04-11

**Authors:** Tingting Chen, Kaihang Wang, Xin Chi, Lizhi Zhou, Jiajia Li, Liqin Liu, Qingbing Zheng, Yingbin Wang, Hai Yu, Ying Gu, Jun Zhang, Shaowei Li, Ningshao Xia

**Affiliations:** 1 State Key Laboratory of Molecular Vaccinology and Molecular Diagnostics, School of Life Sciences, Xiamen University, Xiamen, 361102 China; 2 National Institute of Diagnostics and Vaccine Development in Infectious Disease, School of Public Health, Xiamen University, Xiamen, 361102 China

**Keywords:** Outer membrane protein F, Bacterial surface display, Viral epitope, Genome editing

## Abstract

**Background:**

Bacterial surface display systems were developed to surface expose heterologous proteins or peptides for different applications, such as peptide libraries screening and live bacterial vaccine design. Various outer membrane proteins, such as outer membrane protein A (OmpA), OmpC and outer membrane pore protein E precursor (PhoE), have been used as carriers for surface display, fused to the proteins or peptides of interest in Gram-negative bacteria. Here, we investigated the utility of constitutively expressed OmpF for the display of foreign immune epitopes on the *Escherichia coli* cell surface and then compared it with plasmid-induced expression of OmpF and OmpC.

**Results:**

Enhanced expression of OmpF was linked to a mutation in the OmpF promoter sequence. This mutation rendered OmpF an ideal carrier protein for the enriched display of a target of interest on the bacterial surface. To this end, we grafted two peptides, harboring important epitopes of the hepatitis B virus (HBV) S antigen and human papilloma virus (HPV) L2 protein, onto *OmpF* of *E. coli* by genome editing. The resultant fused OmpF proteins were constitutively expressed in the edited *E. coli* and purified by membrane component extraction. The epitope that displayed on the bacterial surface was verified by SDS-PAGE, western blotting, flow cytometry, and immunoelectron microscopy of the intact bacteria. We further compared this constitutive expression with plasmid-induced expression of OmpF and OmpC in bacterial cells using the same methods for verification. We found that plasmid-induced expression is much less efficient than constitutive expression of OmpF from the bacterial genome.

**Conclusions:**

Enhanced expression of OmpF in a plasmid-independent manner provides an amenable way to display epitopes on the bacterial surface and sheds light on ways to engineer bacteria for biotechnological applications.

**Electronic supplementary material:**

The online version of this article (10.1186/s12934-019-1120-2) contains supplementary material, which is available to authorized users.

## Background

Microbial cell surface display systems were first described in 1986 by Freudl et al. [1] and Charbit et al. [2] as a way to expose heterologous proteins or peptides on the surfaces of micro-organisms, including bacteria, viruses, and phages [3]. Since then, this novel technology has been developed and investigated in several applications, including biocatalysis [4, 5], peptide library screening [6, 7], and live bacterial vaccine production [8, 9]. Using recombinant DNA technology, heterologous antigens can be incorporated onto the cell surface via fusion with bacterial surface proteins via anchoring motifs or efficient signaling peptides. Outer membrane proteins (OMPs) [10], lipoproteins [8] and autotransporters [11] are frequently used as carriers for Gram-negative bacteria surface display.

OMPs of Gram-negative bacteria are ideal carrier proteins to present foreign proteins or peptides on the bacterial cell surface [10]. OMPs span the membrane several times. They comprise a common structural motif of a beta-barrel, which contains a variable number of transmembrane anti-parallel beta-strands connected with short periplasmic turns and long external loops [12]. These protruding loops are the permissive sites that can harbor foreign peptides or protein fragments for surface exposure. Different OMP carriers, such as *Escherichia coli* OmpA [13], OmpC [14] and the outer membrane protein pore E precursor (PhoE) [15], offer different display systems; for example, the Hepatitis B virus core 149 antigen can be displayed on the outer membrane of bacterial ghosts (BGs) via OmpA fusion [16].

OmpF—one of the major outer membrane porin proteins in *E. coli*—allows for the passive diffusion of small hydrophilic molecules across the outer membrane. Three OmpF molecules combine to create a pore on the outer membrane of *E. coli*, with each subunit forming a beta-barrel consisting of 16 transmembrane antiparallel beta-strands. The beta-barrels are connected by eight internal loops and eight external loops surrounding a large channel [17]. In general, the amino acid sequences of the external loops are less conserved, and thus might tolerate insertions. The expression of OmpF and OmpC is transcriptionally regulated by the phosphoprotein OmpR, which can be phosphorylated by the osmotic signal sensing protein, EnvZ [18]. The EnvZ–OmpR system is one of the most well-characterized two-component systems (TCS) of *E. coli*. It functions by responding to changes in environmental osmolality [19]: with increasing osmolality, *OmpF* expression is repressed whereas *OmpC* expression is enhanced. In more concrete terms, in response to increasing external osmolality, EnvZ is phosphorylated, resulting in the phosphorylation of OmpR, which binds to the upstream regulatory regions of the porin genes that encode for OmpF and OmpC. Under low-osmolarity conditions, *ompF*, with its high affinity OmpR-binding sites, is transcribed even when activated OmpR is scarce. However, when the concentration of phosphorylated OmpR increases, the additional binding of OmpR to *ompC* upstream regulatory sites increases *ompC* transcription and *ompF* repression. Although OmpF has not been used in bacterial surface display systems, the OmpF protein or fragment has been used previously as a fusion partner for the excretion of human β-endorphin into the culture medium during high-cell-density cultivation [20, 21], and to yield high quantities of the full-length membrane protein human receptor activity-modifying protein 1 (RAMP1) [22].

In the present study, we found that OmpF expression can be substantially enhanced in an engineering bacterial strain, ER2566, when an inadvertent mutation is introduced into its promoter, hereafter referred to as ER0808. Using this modified strain, we constructed a novel *E. coli* surface display system by engineering OmpF with two important epitopes, “TSTGPCKTCTTPA” from hepatitis B surface antigen (HBsAg) and “QLYKTCKQAGTCPPDII” from human papillomavirus (HPV) L2 [23]. We then compared this constitutive expression of OmpF with plasmid-induced expression of OmpF and OmpC and found constitutively expression OmpF to be more efficient. Overall, we show that OmpF can serve as a good endogenous carrier protein in constitutive cell-surface display.

## Results

### Point mutation upstream results in constitutive expression of OmpF

**Fig. 1:**
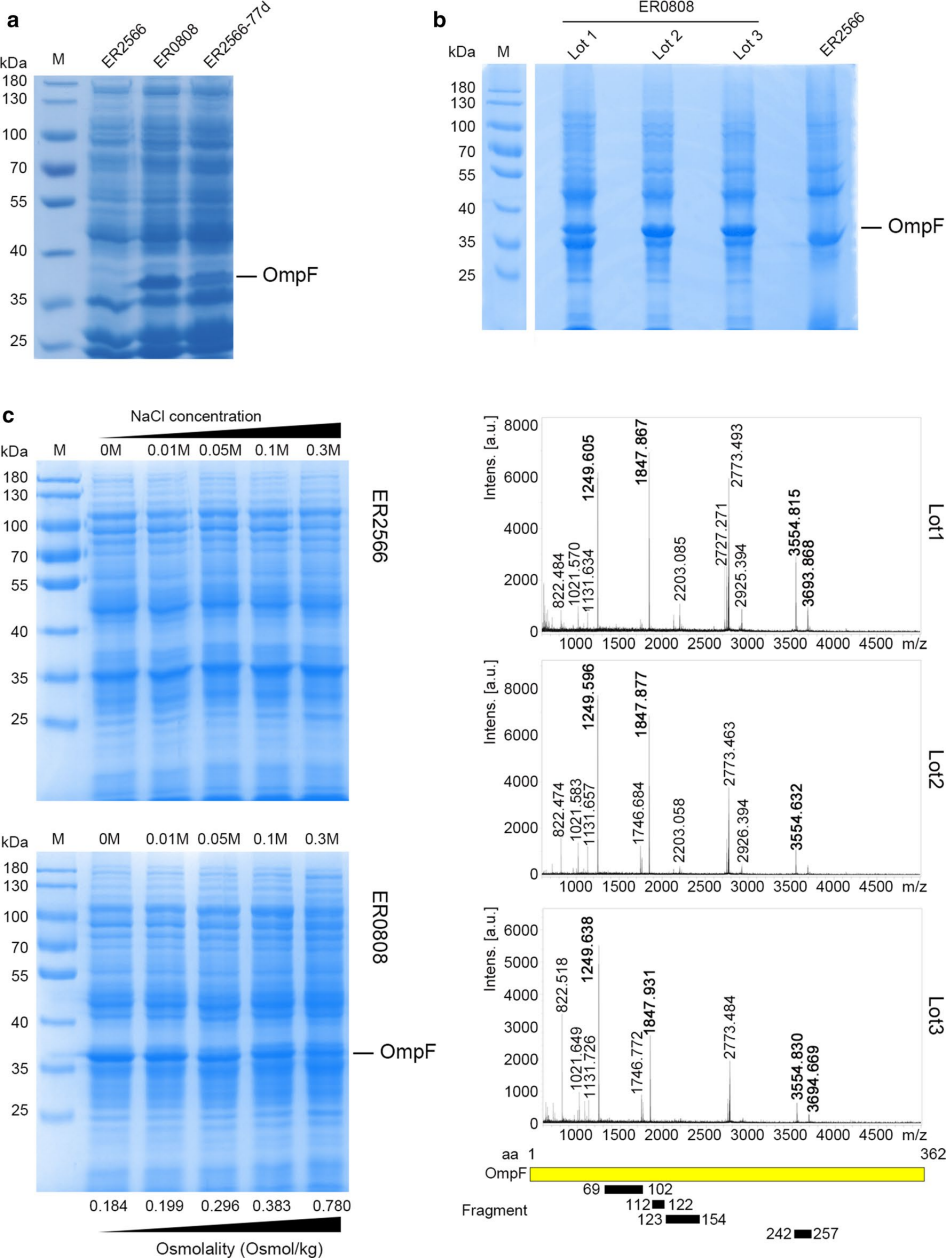
Enhanced and constitutive expression of OmpF in ER0808. **a** SDS-PAGE profile of whole bacterial lysate shows an abundant expression of OmpF. Samples (10 μL), with an approximately equal amount of cell lysate, were loaded in each lane. *M* marker. **b** Top: SDS-PAGE profile shows 3 sets of protein bands excised from Coomassie blue-stained SDS-PAGE gels (black arrows) for further analysis. Samples (10 μL) with an approximately equal amount of cell lysate, were loaded in each lane, and a rough estimate for the amount of protein in each band was ~ 10 µg. Lower: matrix-assisted laser desorption/ionization (MALDI)-time-of-flight/time-of-flight mass spectrometry of the suspected OmpF protein band from ER0808. The observed trypsin-cleaved peptides that match theoretical ones of OmpF are denoted as black bars distributed in an OmpF schematic map according to their amino acid locations, which account for a sequence coverage of 26%. **c** OmpF expression profiles in the ER0808 stain and its original ER2566 strain under a variety of osmolality presets for bacteria growth

In the *E. coli* porin regulon, OmpF and OmpC are regulated by the OmpR–EnvZ system in response to osmolarity: OmpC is preferentially produced in high osmolarity medium, such as LB medium, whereas OmpF is repressed [19]. We inadvertently identified an abundance of protein at ~ 37 kD in the SDS-PAGE profile from a culture of the engineering ER2566 *E. coli* strain that was not present in regular cultures. We hereafter refer to this mutant strain as ER0808 (Fig. 1a). The protein bands in question were excised from Coomassie blue-stained SDS-PAGE gels from 3 sets of ER0808 lysates (upper panel, Fig. 1b), treated with trypsin and then subjected to TOF/TOF MS analysis. We then undertook a peptide mass fingerprint search on the resultant major MS peaks (lower panel, Fig. 1b) using the Mascot tool (http://www.matrixscience.com). The protein abundant in ER0808 but not in ER2566 was identified as OmpF. This was confirmed across 4 unique trypsin-digested peptides that exactly matched the theoretical enzymatic fragments of the bacterial OmpF protein (Table 1). Interestingly, the constitutive expression of OmpF in the ER0808 strain was slightly repressed under high (0.3 M NaCl) osmolarity conditions even though OmpF expression was superficially abundant in the bacterial lysates at all osmotic conditions. However, unlike the high OmpF expression at low osmolarity in the K12 strains ([24]), we did not observe OmpF expression in ER2566 even under the lowest osmolarity tested (0 M NaCl) (Fig. 1c).

**Table 1:** Observed molecular weights of OmpF peptide fragments digested with trypsin and measured by liquid chromatography-mass spectrometry

Fragment	Peptide sequence	Theoretical M.W. (Da)	Observed M.W. (Da)		
			Lot 1	Lot 2	Lot 3
1	YADVGSFNYGR	1248.541	1249.605	1249.596	1249.638
2	YDANNIYLAANYGETR	1846.849	1847.867	1847.877	1847.931
3	NYGVVYDALGYTDMLPEFGGDTAYSDDFFVGR	3553.566	3554.815	3554.632	3554.830
4	GETQINSDLTGYGQWEYNFQGNNSEGADAQTGNK	3693.573	3693.868		3694.669

**Fig. 2:**
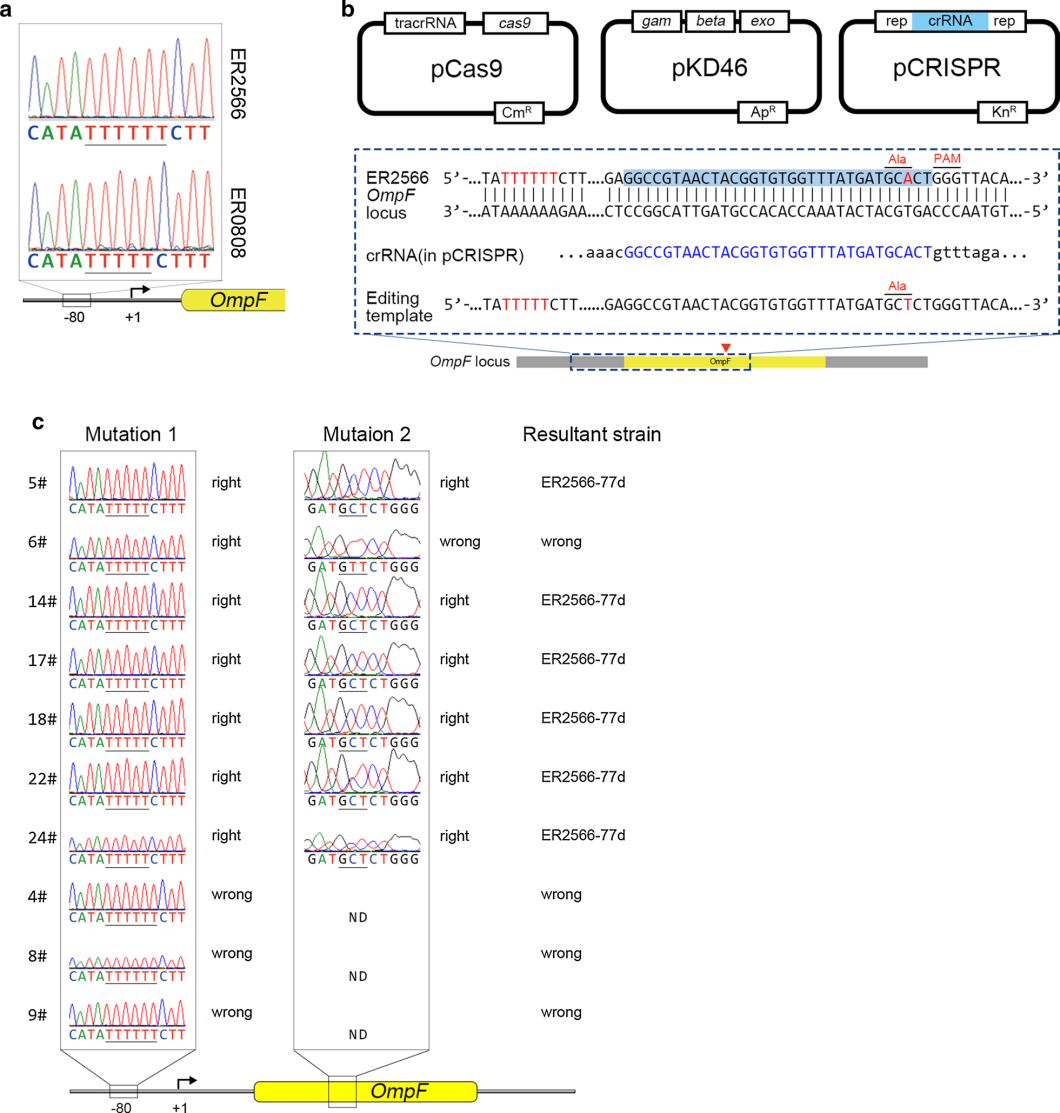
Point mutation in the promoter resulted in enhanced expression of *OmpF*. **a** Sequences of the *OmpF* promoter − 80 region. The black box indicates the mutation site that differs between the normal ER2566 strain and the OmpF-constitutive-expression strain ER0808. **b** Point mutation strategy utilizing CRISPR/Cas9-coupled lambda Red recombineering. The sequence corresponding to the *ompF* protospacer is shown in blue, and the sequence corresponding to the − 77 locus is shown in red. “Rep” corresponds to direct repeat sequences necessary for crRNA processing that flank the targeting spacer. **c** Representative sequencing results of the *OmpF* promoter at the − 80 region from edited ER2566 colonies. Of 24 independent colonies randomly picked for editing confirmation, 6 colonies (5#, 14#, 17#, 18#, 22# and 24#) harbored the expected silent Ala130 mutation (corresponding to GCA to GCT) and the 1-bp deletion of a thymidine at the − 77 locus. One colony (6#) suffered a wrong mutation of Ala130 to Val130 (GCA to GTT), and the remaining 17 colonies had no 1-bp deletion of a thymidine. Sequences for 4#, 8# and 9# are shown

Using DNA sequencing, we found a 1-bp deletion of a thymidine in a short string of T residues in the upstream ER0808 *OmpF* region, located between − 77 and − 83 bp from the start site of transcription (Fig. 2a). This so-called *cis*-regulatory mutation in the MC4100 strain is believed to regulate the OmpR binding structure of the promoter region and result in an OmpF-constitutive phenotype [24]; this likely explains the overexpression of OmpF in high osmolarity LB medium in our cultures.

To verify our hypothesis that the *cis*-regulatory mutation of OmpF triggers its constitutive expression in ER0808, we generated the pCRISPR-porin and editing template B1 to introduce a single base deletion in the ER2566 *OmpF* − 77 locus using the CRISPR-Cas9 system, with a silent mutation in Ala130 (GCA to GCT) upstream of PAM (Fig. 2b). Sequencing showed a single deletion in the exact upstream locus and a silent mutation in Ala130 (Fig. 2c). Thus, the resultant genome-edited strain was named ER2566-77d. As calculated by the ratio of correct editing colonies, the editing efficiency of CRISPR approach was 25%. SDS-PAGE of whole bacterial total protein (TP) showed a conspicuous protein band at ~ 37 kDa, similar to that produced by ER0808 (Fig. 1a). These results confirmed that the point mutation on the *cis*-regulatory region resulted in abundant constitutive expression of OmpF in ER0808.

### Cell surface display system construction using genome editing techniques

**Fig. 3:**
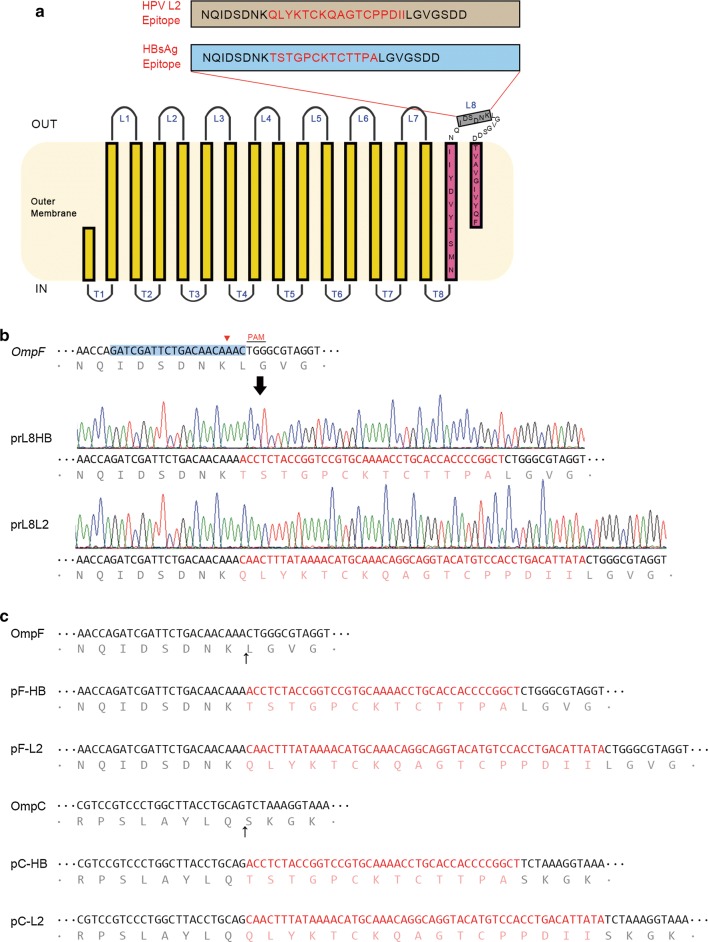
Design of the fusion proteins and genome editing for the display system. **a** Structure of OmpF and the amino acid sequences of the epitope inserts (red). Epitope peptide was designed to be integrated into OmpF loop 8 for surface display. **b** Genome editing for insertion of the epitopes into *OmpF* in *E. coli*. The protospacer for CRISPR system target is shaded in blue in *OmpF.* DNA sequencing was used to verify the editing of the bacterial genome. The translated amino acid sequences are consistent with the epitope sequences (E6F6, TSTGPCKTCTTPA; 14H6, QLYKTCKQAGTCPPDII). **c** Genetic sequences and corresponding amino acid sequences of epitope inserts in constructed plasmids pF-HB, pF-L2, pC-HB, and pC-L2. The original and epitope-grafted sequences of OmpF from the ER2566 strain and OmpC from the K12 strain are shown. Black arrows indicate the sites of epitope insertion

Based on the structure of OmpF and the genome PAM analysis, loop 8 of OmpF was selected as the insert locus for peptide fusion. Genome editing techniques were used to introduce the exogenous gene sequence into loop 8 between Lys345 and Leu346 of OmpF. Two important virus antigen epitopes, HBsAg peptide “TSTGPCKTCTTPA” (recognized by murine monoclonal antibody E6F6) and HPV16 L2 peptide “QLYKTCKQAGTCPPDII” (recognized by murine monoclonal antibody 14H6), were used as model inserts to develop the system (Fig. 3a). Our previous studies have shown that these epitopes are important vaccine targets for antigen design.

We generated the pCRISPR-L8 plasmid harboring a specific crRNA that would guide Cas9 cleavage of the OmpF loop 8 locus gene. The B2 and B3 editing templates, containing the epitope sequence and flanking homology arms, were constructed for homologous recombination. The ER0808 strain harboring pCas9 and pKD46 [25] was co-transformed with the pCRISPR-L8 plasmid and the B2 or B3 editing template, and the epitope sequence was introduced into the OmpF gene special site after recombination, constructing the recombinant strains, ER0808-prL8HB and ER0808-prL8L2. DNA sequencing results showed that the insertion element was consistent with our design (Fig. 3b).

### OmpF-mediated surface display of viral epitope in *E. coli*

In the literature, OMPs are often used to construct a bacterial surface display system by plasmid-induced expression [14, 15]. Next, we sought to compare our OmpF constitutive display system with plasmid-induced expression of OmpF and OmpC.

OmpF and OmpC proteins were expressed bearing both E6F6 and 14H6 epitopes by engineered pTO-T7 expression plasmids: pF-HB and pF-L2 (OmpF); and pC-HB and pC-L2 (OmpC). In pF-HB and pF-L2 plasmids, the epitopes were grafted to loop 8 of OmpF as per the aforementioned genome editing design, whereas, for pC-HB and pC-L2, the epitopes were inserted into loop 7 of OmpC, as described previously [14] (Fig. 3c). Plasmids were transformed into ER2566 cells for over-expressions of fusion proteins.

**Fig. 4:**
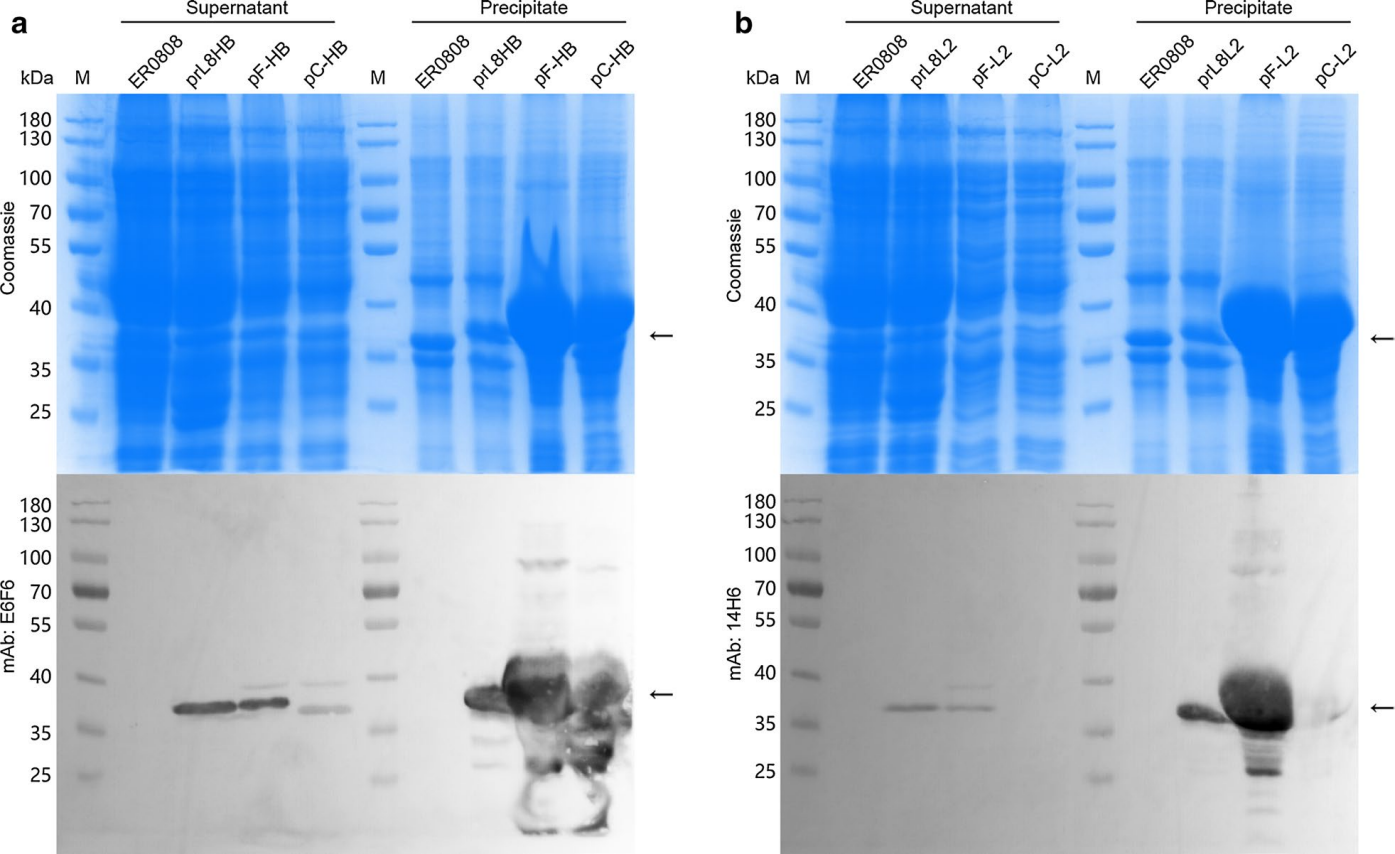
Analysis of fusion protein expression. SDS-PAGE and western blotting analysis of the bacterial lysates of **a** bacteria ER0808, ER0808-prL8HB, ER2566(pF-HB) and ER2566(pC-HB), and **b** bacteria ER0808, ER0808-prL8L2, ER2566(pF-L2) and ER2566(pC-L2). Black arrows indicate the fusion proteins

**Fig. 5:**
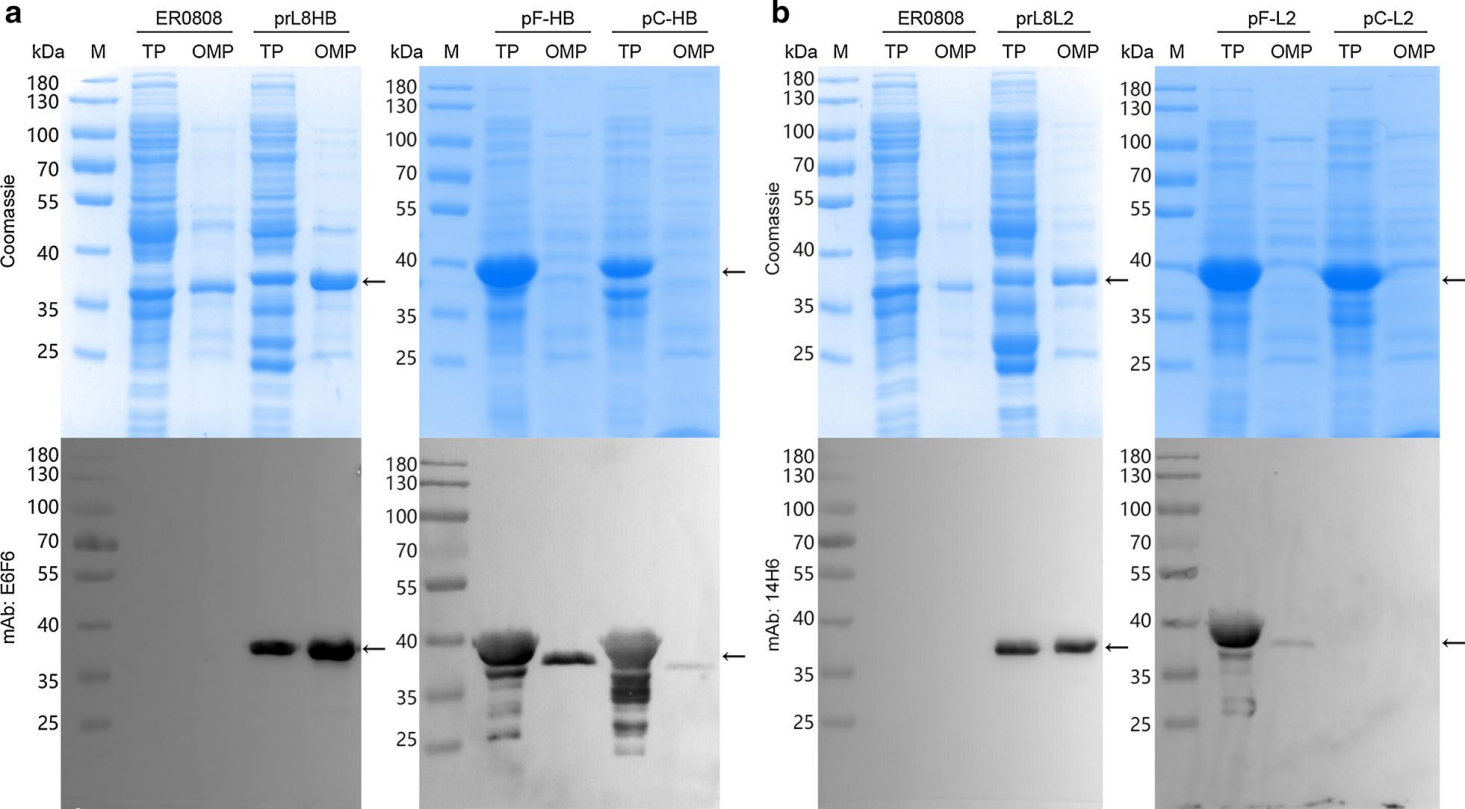
Identification of fusion protein expression and purification. SDS-PAGE and western blotting of total proteins (TP) and OMPs prepared from equal biomass of ER0808 and **a** ER0808-prL8HB, ER2566(pF-HB) and ER2566(pC-HB) or **b** ER0808-prL8L2, ER2566(pF-L2) and ER2566(pC-L2). Black arrows indicate the fusion proteins. Samples (10 µL) with an equivalent amounts of cell lysate, were loaded

The supernatants and precipitants of the bacteria lysates were analyzed by SDS-PAGE and western blotting with E6F6 and 14H6 mAbs. Both OmpF and OmpC were abundantly expressed by plasmid induction in bacteria ER2566(pF-HB), -(pF-L2), -(pC-HB) and -(pC-L2) as compared with constitutive expression from the bacterial genome in bacteria ER0808-prL8HB and ER0808-prL8L2. Most of the proteins from plasmid expression were found in the cell debris, which were thought to have aggregated as inclusion bodies (Fig. 4). On the other hand, the outer membrane fractions were extracted from both *E. coli* ER0808-prL8HB and ER0808-prL8L2 bacteria and showed reactive with mAb E6F6 and 14H6, respectively, in western blotting (Fig. 5a, b, left panel), indicating the localization of the peptides to the outer membranes of bacteria containing the OmpF fusion protein. In contrast, much less OmpF and OmpC fusion proteins produced through plasmid-induced expression could be extracted from the bacterial membranes (Fig. 5a, b, right panel), suggesting that constitutive expression may provide a beneficial way to express OMPs for bacterial surface expression.

**Fig. 6:**
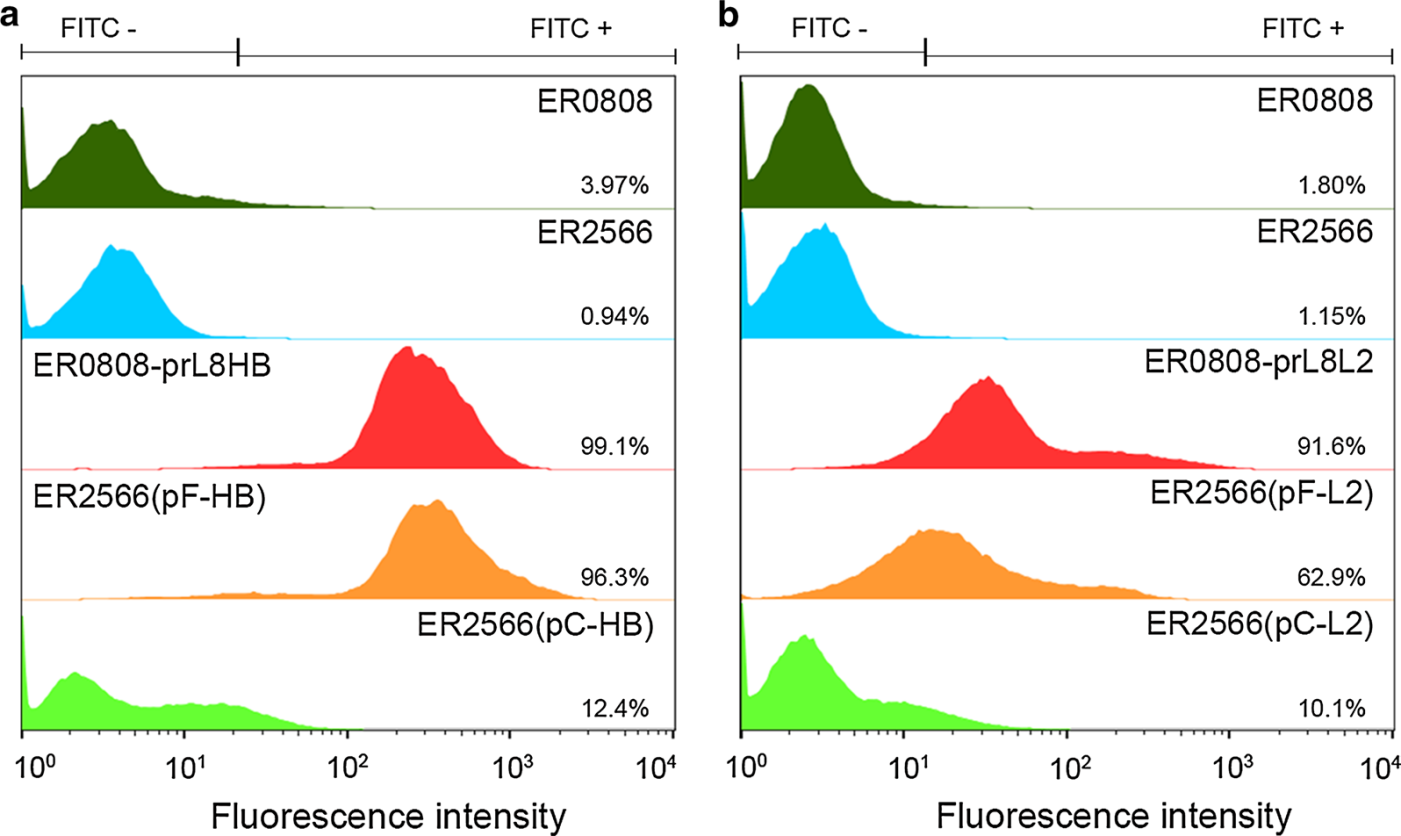
Flow cytometry of recombinant bacteria. E6F6 epitope display strains (**a**) and E6F6 epitope display strains (**b**) were analyzed with E6F6 or 14H6 as the primary antibodies and FITC-conjugated secondary antibodies in flow cytometer. ER2566 and ER0808 served as negative control. The results were illustrated as histogram mode and positive cell ratios were indicated

Next, we performed flow cytometry to quantify the number of mAb E6F6- and 14H6-reactive cells. Bacterial cells were incubated with corresponding primary mAbs followed by the appropriate secondary FITC-conjugated antibodies, and then prepared for flow cytometry. ER2566 and ER0808 strains served as the controls. As indicated in Fig. 6, ER0808-prL8HB and ER0808-prL8L2 cells, which constitutively expressed OmpF, demonstrated a higher fluorescence intensity than the control, and we attributed this to mAb E6F6 or 14H6 reactions, with 99.1% and 91.6% positive cell counts, respectively. Yet in plasmid-induced OmpF expression, ER2566(pF-HB) and ER2566(pF-L2) cells exhibited about 96.3% and 62.9% positive cell counts, respectively, which suggested that OmpF bearing E6F6 and 14H6 epitopes can translocate to the bacterial surface; although, few fusion proteins could be extracted from the bacterial membrane. In contrast, OmpC-displaying strains, ER2566(pC-HB) and ER2566(pC-L2), showed very low positive cell counts (12.4% and 10.1%, respectively). Overall, the constitutive expression-based OmpF display system generates a higher positive cell ratio than the plasmid-induced expression system due to more abundant membrane-resident OmpF. In addition, plasmid-induced OmpF leads to a significantly higher surface decoration cell ratio than does plasmid-induced OmpC.

**Fig. 7:**
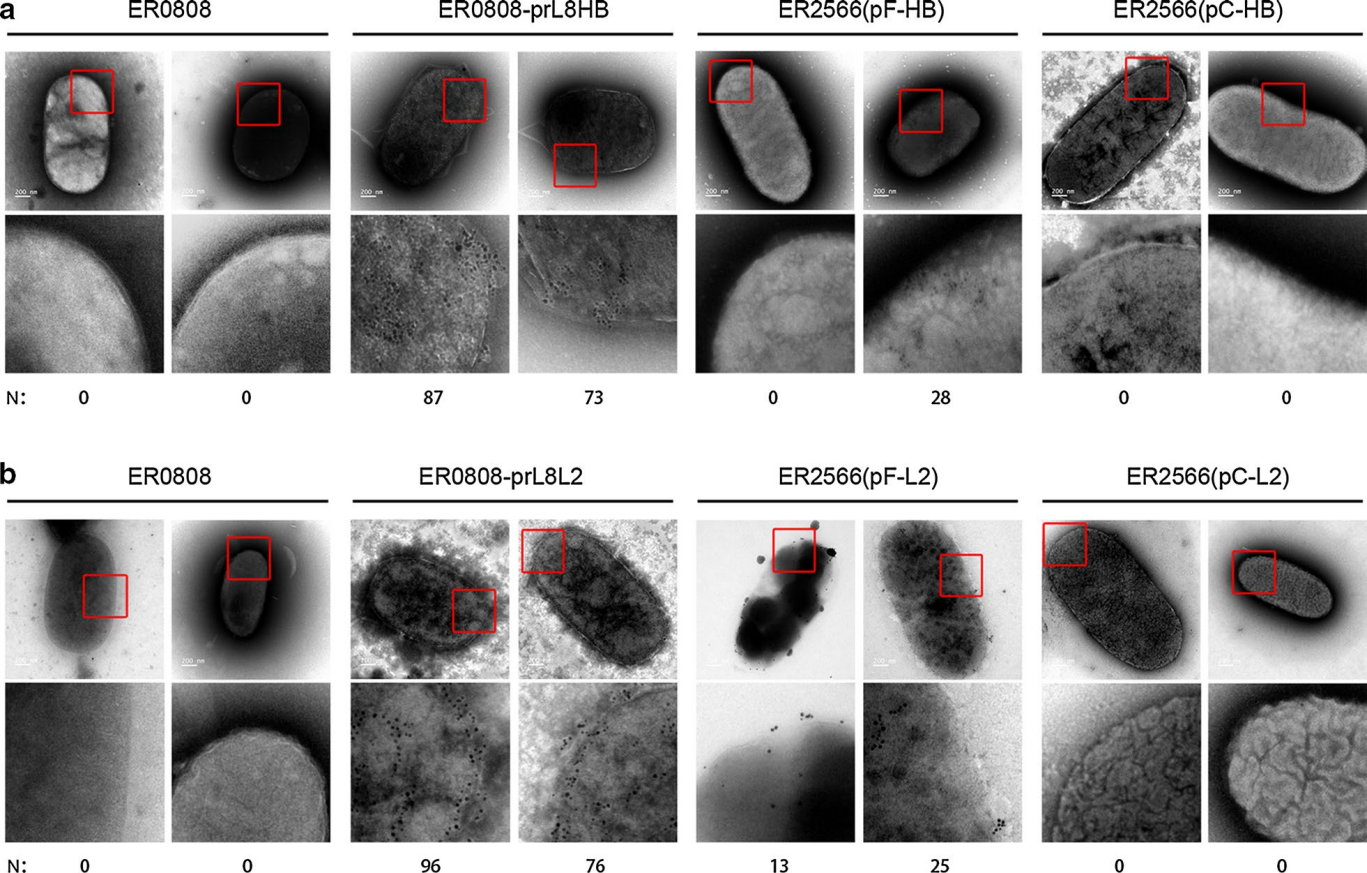
Immunoelectron microscopy of recombinant bacteria. **a** Electron microscopy (EM) images of ER0808 cells, ER0808-prL8HB cells, ER2566-pF-HB cells and ER2566-pC-HB cells, as indicated by the gold-conjugated E6F6 (set A, top panel) at ×49,000 magnification. **b** EM images of ER0808 cells, ER0808-prL8L2 cells, ER2566-pF-L2 cells and ER2566-pC-L2 cells as indicated by gold-conjugated 14H6 (set **b**, top panel) at ×49,000 magnification. For clarity, close-up views of the top panels are shown; red boxes indicate the magnified regions. The curve of some images below is adjusted to better visualize the gold particles by Photoshop software. Number of gold particles on the bacterial surface presented in the close-up views was counted by Multi-point tool of ImageJ software (http://rsb.info.nih.gov/ij/), and is shown below the images as “N”

Finally, we used immunoelectron microscopy to confirm the outer membrane display of the epitopes of interest. Murine monoclonal antibodies E6F6 and 14H6 conjugated to 10-nm gold particles were bound to the intact bacteria. Gold particles were specifically visualized on the surfaces of the epitope-bearing bacteria ER0808-prL8HB and ER0808-prL8L2, which constitutively expressed OmpF fusion proteins. In contrast, few particles could be visualized on the surface of bacteria ER2566(pF-HB) and ER2566(pF-L2) of plasmid-induced OmpF fusion proteins, and no gold particles were observed on the surface of bacteria ER2566(pC-HB) and ER2566(pC-L2) expressing OmpC (Fig. 7a, b). These results are consistent with those in Fig. 6. Taken together, we show that constitutive expression of OmpF can more efficiently coat the surface of *E. coli* with target epitopes than plasmid-induced OmpF or OmpC expression methods.

## Discussion

This study describes the display of viral epitope peptides on the surface of genome-edited *E. coli* cells. It is a novel surface display system that employs OmpF as a carrier protein by sandwich fusion to incorporate short peptides in surface-accessible regions of OmpF. In traditional display systems, recombinant plasmids are transcriptionally controlled by an inducible promoter, which is capable of achieving a high yield of fusion protein as compared to an endogenous carrier protein, such as OMP. Overexpressed OmpF was identified due to a mutation in the promoter, thus offering an ideal carrier protein system that is independent of its exogenous inducible expression. Genome editing technology was then employed to generate sandwich fusion of the viral epitope peptide for further constitutive expression and surface display. Further tests, such as BGs construction and immunization, are needed to evaluate the immunogenicity of the recombinant bacteria.

Although constitutive expression by the bacterial genome generated a lower expression yield, our analysis of the protein extracts from the outer membrane using flow cytometry and immunoelectron microscopy indicated that constitutive expression more efficiently displayed OmpF on the bacterial surface than the plasmid-induced system. This may be because the constitutive expression of OmpF is an innate function to establish porin array on its membrane for material transfer across the cellular membrane. The other reason would be the single mutation in the promoter of OmpF, which enhances the expression yield, as shown in our study (Fig. 1a) and elsewhere [24].

In K12 strains, such as MC4100-derived JMS750 [24] and N99 [26], the expression levels of OmpF and OmpC are transcriptionally regulated by osmolality, with high osmolality leading to a downregulation in OmpF and an upregulation of OmpC. However, OmpF expression in ER2566 remained low at all of the tested osmolarity conditions, and ER0808 conferred a high OmpF expression level overall; albeit, levels were slightly lower under high osmolality (Fig. 1c). Of note, ER2566 belongs to the BL21 strain and does not synthesize OmpC due to a partial deletion of the *ompC* gene in its genome; it is possible that this might lead to a differential control of OmpF expression, and that perhaps OmpC and OmpF mutually establish an osmotic balance for bacterial physiology [19]. Nonetheless, the high expression level of OmpF in ER0808 under regulation of the inadvertently mutated promotor improves the epitope display efficiency for *E. coli*-based vaccine design.

Over the past few years, the bacterial ghost (BG) system has provided a new platform for vaccine development [27]. BGs are empty Gram-negative bacterial envelopes produced by the controlled expression of the cloned lysis gene E from bacteriophage PhiX174, which possesses all the immunostimulatory surface properties of the original host strain without the infectious threat [27, 28]. Using bacterial surface display technology, foreign antigens in the form of proteins or epitope peptides can be presented on the outer membrane of BGs, which provides an excellent vehicle system for antigen delivery [16]. Our results showed that an OmpF-based surface display platform can be used to decorate the surface of *E. coli* with viral epitopes, in which the antigen was endogenously and constitutively expressed. Furthermore, our study outlines a genome editing approach for the constitutive expression of the protein in situ from the bacterial genome. Indeed, the OmpF expression regulation and membrane localization described here could be extrapolated to other membrane proteins to express them in the correct native conformation, which is prerequisite for immunogen application. Thus, our novel OmpF system holds great strategic potential for the design of BG-based vaccines.

## Conclusions

We developed a new bacterial surface display system based on OmpF as a carrier protein. In this system, OmpF was abundantly overexpressed under the regulation of its mutated promoter, and was designed to incorporate a heterologous peptide within its surface loops. Two important viral epitope peptides were successful displayed on the surface of *E. coli* cells. Our display system provides an amenable way to engineer bacteria for biotechnological applications.

## Methods

### Bacterial strains, plasmids, and growth conditions

The *E. coli* strain ER0808 is derived from a mutation strain in ER2566 strain (New England Biolabs, Beverly, USA) and preserved in our laboratory.

Plasmid pCRISPR (Addgene plasmid # 42875) and pCas9 (Addgene plasmid # 42876) were gifts from Luciano Marraffini (The Rockefeller University, New York, NY, USA). Plasmid pKD46 was purchased from Coli Genetic Stock Center (Yale University, New Haven, CT, USA). Plasmid pTO-T7 [29] was preserved in our laboratory.

Luria–Bertani (LB) broth was used for regular culture of cells. To verify whether OmpF expression is transcriptionally regulated under the environmental osmolality, we measured the OmpF expression profiles in the ER0808 stain and its original ER2566 strain under a variety of osmolality presets for bacteria growth, according to the protocol by Jubelin et al. [30]. Briefly, the basic culture medium was twofold diluted in M63 medium containing 0.2% glucose. Then various final concentrations (0.01 M, 0.05 M, 0.1 M or 0.3 M) of NaCl were prepared in basic medium to generate an osmotic gradient for OmpF expression in ER0808 and ER2566. Osmolarity was measured with a SMC30C osmometer (Tianjin Tianhe, Tianjin, China). Ampicillin (100 μg/mL), kanamycin (25 μg/mL), and chloramphenicol (250 μg/mL) were added, as needed. All bacterial strains were grown at 37 °C, and those strains with plasmid transformation were induced at an OD_600 nm_ of 0.6 by the addition of a final concentration of 0.4 mM isopropyl-beta-d-1-thiogalactopyranoside (IPTG) for 10 h at 24 °C.

### DNA isolation, manipulation, and plasmid construction

The sequences of the primers used in this study are listed in Table 2. Splicing overlap extension (SOE) PCR was introduced to generate donor DNA as the genome editing template, which comprised the exogenous sequence with about 400-bp flanking homologous arms. To generate the ER2566-77d strain, which has a single base deletion in the ER2566 *OmpF* − 77 locus, the 626-bp editing template (named as B1) was amplified with primers A01/A02 from ER0808 genome DNA, containing a thymidine deletion in the − 77 locus and a silent mutation in the Ala130 (GCA to GCT) site, in front of the protospacer adjacent motif (PAM). To generate loop-insertion donor DNA, the editing template (B2) was amplified with primers A03/A04 and primers A05/A06 containing the HBsAg epitope sequence (ACCTCTACCGGTCCGTGCAAAACCTGCACCACCCCGGCT) from ER0808 genomic DNA; alternatively, the editing template (B3) was amplified with primers A03/A07 and primers A08/A06 containing the HPV16 L2 epitope sequence (CAACTTTATAAAACATGCAAACAGGCAGGTACATGTCCACCTGACATTATA).

**Table 2:** Primer sequences used in this study

Primer	Sequence (5′ → 3′)
A01	CTTAAATTTTACTTTTGGTT
A02	AGCATATCGGTGTAACCCAGAGCATCATAA
A03	CTTTGGTATCGTTGGTGCTT
A04	GGTGCAGGTTTTGCACGGACCGGTAGAGGTTTTGTTGTCAGAATCGATCT
A05	GGTCCGTGCAAAACCTGCACCACCCCGGCTCTGGGCGTAGGTTCAGACGA
A06	GCTGGTCAGTACCGGGGTTT
A07	AGGTGGACATGTACCTGCCTGTTTGCATGTTTTGTTGTCAGAATCGATCT
A08	AAAACATGCAAACAGGCAGGTACATGTCCACCTCTGGGCGTAGGTTCAGA
A09	TTTAAGAAGGAGATATACATATGATGAAGCGCAATATTCT
A10	TTGTTAGCAGCCGGATCTCAGAACTGGTAAACGATACCCA
A11	TTTAAGAAGGAGATATACATATGAAAGTTAAAGTACTGTCCCTCCTGGTCCCAGCTCTG
A12	GGTGCAGGTTTTGCACGGACCGGTAGAGGTCTGCAGGTAAGCCAGGGACG
A13	GGTCCGTGCAAAACCTGCACCACCCCGGCTTCTAAAGGTAAAAACCTGGG
A14	TTGTTAGCAGCCGGATCTCAGAACTGGTAAACCAGACCCA
A15	AGGTGGACATGTACCTGCCTGTTTGCATGTTTTATAAAGTTGCTGCAGGTAAGCCAGGGA
A16	AAAACATGCAAACAGGCAGGTACATGTCCACCTGACATTATATCTAAAGGTAAAAACCTG
A17	CATATGTATATCTCCTTCTT
A18	TGAGATCCGGCTGCTAACAA

The fragment (nt 361–392) of OmpF sequence: GGCCGTAACTACGGTGTGGTTTATGATGCACT and OmpF (nt 1000–1036) sequence: GACTACATCATCAACCAGATCGATTCTGACAACAAAC, both followed by a PAM in the ER0808 genome, were synthesized de novo and ligated into the product with BsaI-digested pCRISPR plasmid to generate an OmpF-targeted sgRNA vector, as reported before [31], thereby constructing the pCRISPR-porin and pCRISPR-prL8 plasmids, respectively.

To construct plasmids for the expression of OmpF fusion proteins (named plasmid pF-HB and pF-L2), the OmpF genes with epitope inserts (B4 and B5) were amplified from the ER0808-prL8HB and ER0808-prL8L2 recombinant strains with primers A09 and A10, respectively. To construct the expression plasmid (pC-HB and pC-L2) for the OmpC fusion proteins, DNA fragments (B6 and B7) were amplified with primers A11/A12 and primers A13/A14 containing the sequence encoding the HBsAg epitope from K12 genomic DNA; the DNA fragments (B8 and B9) were amplified with primers A11/A15 and primers A16/A14 containing the sequence encoding the HPV16 L2 epitope from K12 genomic DNA. Finally, 4 sets of DNA fragments—B4, B5, (B6 and B7), (B8 and B9)—were individually ligated into products that were amplified with primers A17/A18 and using a nonfusion pTO-T7 expression vector as a PCR template, as per the “Gibson Assembly” method [32]. All of the resultant plasmids were confirmed by DNA sequencing and transformed into ER2566 by the CaCl_2_ method for protein expression. The full OmpF nt sequence with highlighted protospacers; the sequences of HDR repair templates B1, B2, and B3; and the sequences of DNA fragments B4, B5, B6, B7, B8, B9 are listed in Additional file 1: Text S1.

### CRISPR/Cas9-coupled lambda Red recombineering

ER0808 or ER2566 cells harboring pCas9 and pKD46 were used as the host strain for CRISPR/Cas9 recombineering and prepared for electro-transformation. Cultures for electroporation were grown at 30 °C. Arabinose (10 mM final concentration) was added to the culture at an OD_600_ of 0.6 for 1 h to induce the lambda Red (λ-Red) recombineering operon of pKD46. Immediately after induction, cells were harvested and prepared as competent cells, according to a previously published protocol [33]. For genome editing, 300 ng pCRISPR plasmid and 500 ng editing template were electro-transformed into ER0808 (pCas9-pKD46) or ER25660 (pCas9-pKD46) competent cells. Electroporation was carried out in a prechilled 0.1-cm gap sterile electroporation cuvette using a BioRad GenePulser II under the following parameters: 2500 V, 200 Ω, 25 μF. The expected time constant should be 4.5 to 4.9 ms. The correct recombinant colony was confirmed by sequencing. The strains into which the OmpF gene was inserted with the HBsAg epitope sequence or the HPV L2 epitope sequence were named as ER0808-prL8HB and ER0808-prL8L2, respectively.

### Protein assay

An overnight culture of the transformant was grown in LB medium at 37 °C or induced by IPTG, as described in “Bacterial strains, plasmids, and growth conditions” section. Cells harvested from 1 mL bacterial culture with OD_600 nm_ of about 2.0 were resuspended in 100 µL phosphate buffer saline, pH 7.4 (PBS), boiled in loading buffer for 10 min at 100 °C, and centrifuged at 13,000 rpm for 10 min to obtain the total protein (TP) samples for following SDS-PAGE and Western blotting analysis. In parallel, 500 mL bacterial culture with OD_600 nm_ of about 2.0 was pelleted and resuspended with 10 mL PBS. The supernatant and precipitant samples were prepared from the centrifugation of sonication-treated lysate. To check whether high osmolarity downregulated the expression of OmpF in ER0808, TP samples were prepared from an equal amount of cell lysates from different strains (0.2 mg wet cell weight). Outer membrane fraction samples were prepared based on a temperature-induced phase separation technique from the intact *E. coli* cells. Cells were harvested and then suspended in equal volumes of 10 mM Tris-base buffer (pH 7.4) with 150 mM NaCl and 1% Triton X-114 for 90 min at 4 °C before centrifugation at 10,000 rpm for 10 min at 4 °C. The supernatant was incubated at 37 °C for 15 min and further centrifuged at 3000 rpm for 10 min. The organic phase containing the membrane protein was precipitated with acetone at 4 °C for 40 min, and then centrifuged at 13,000 rpm for 10 min at 4 °C. The precipitated protein was dissolved in water, and boiled in loading buffer for 10 min at 100 °C to obtain the OMP samples.

Protein samples from equal culture densities and volumes were subjected to 10% SDS-PAGE. Prominent proteins were visualized by Coomassie Blue staining, or detected through immunoblotting. For immunoblotting, proteins were electroblotted on nitrocellulose membranes using standard western blotting protocols, blocked with 5% skim milk, and then incubated with 0.2 μg/mL primary mAb E6F6 or 14H6 for 1 h. Membranes were washed and then incubated with 0.2 μg/mL appropriate secondary antibody conjugated to horseradish peroxidase for 1 h, washed again and then visualized using SuperSignal ELISA Pico Chemiluminescent Substrate (PIERCE, Rockford, IL, USA).

### Protein identification by mass spectrometry

Protein bands was excised from Coomassie blue-stained SDS-PAGE gels and subjected to trypsin digestion followed by time-of-flight/time-of-flight mass spectrometry (TOF/TOF–MS) analyses, performed using an Autoflex TOF mass spectrometer (Bruker Daltonics, Bremen Germany). Spectra were analyzed using MASCOT (http://www.matrixscience.com) with peptide mass fingerprinting (PMF), searching in the NCBInr (non-redundant) database.

### Immunoelectron microscopy assay

For immunoelectron microscopy, 100 μL of bacterial culture was harvested and washed twice with 20 mM phosphate buffer (PB) pH 7.0, and then incubated with 10 μL 10-nm gold particle-conjugated E6F6 or 14H6 monoclonal antibodies against HBsAg or HPV L2 (Lab), respectively, for 12 h at 4 °C. The concentration of E6F6-gold was 0.5 mg/mL (weight of antibody/volume) and that of 14H6 was 2.67 mg/mL (weight of antibody/volume). Samples were diluted 1:50 with 20 mM PB pH 7.0, absorbed onto carbon-coated copper grids, blotted dry, and stained with freshly filtered 2% phosphotungstic acid (pH 6.4). Grids were examined under an FEI Tecnai G2 Spirit transmission electron microscope (FEI, Hillsboro, Oregon, US) at an accelerating voltage of 120 kV and then photographed at a nominal 30,000× or 49,000× magnification to capture the whole bacterium in one image.

### Flow cytometry

Different strains of *E. coli* were cultured as described in “Bacterial strains, plasmids, and growth conditions” section. Samples (50 µL) of different strains were added to 500 µL of PBS containing 2% (wt/vol) BSA, 4 µg/mL E6F6 or 6 µg/mL 14H6, and FITC-conjugated anti-mouse IgG antibody (1:2000 or 1:1000, respectively). Mixtures were incubated on ice for 2 h and then centrifuged at 3000×*g* for 5 min to remove unbound antibodies. Pellets were resuspended in 500 µL PBS and processed for flow cytometry (FACS-Cyan ADP, Beckman Coulter). The wash step was omitted to reduce the proportion of broken cells and antibody leakage. Data were analyzed using FlowJo software.

## Additional file

**Additional file 1: Text S1.** Nucleotide sequences of the *OmpF* locus and the DNA fragments used in gene manipulation.
