# Supplementary material for: Construction of a bacterial surface display system based on outer membrane protein F
Source: Microb Cell Fact. 2019 Apr 11;18:70. doi: 10.1186/s12934-019-1120-2 (PMC6458713; doi:10.1186/s12934-019-1120-2)
Supplement: Supplementary file 1 — Additional file 1: Text S1. Nucleotide sequences of the OmpF locus and the DNA fragments used in gene manipulation. [file 12934_2019_1120_MOESM1_ESM.docx]

*OmpF* locus:

AATATGACGGTGTTCACAAAGTTCCTTAAATTTTACTTTTGGTTACATATTTTTCTTTTTGAAACCAAATCTTTATCTTTGTAGCACTTTCACGGTAGCGAAACGTTAGTTTGAATGGAAAGATGCCTGCAGACACATAAAGACACCAAACTCTCATCAATAGTTCCGTAAATTTTTATTGACAGAACTTATTGACGGCAGTGGCAGGTGTCATAAAAAAAACCATGAGGGTAATAAATAATGATGAAGCGCAATATTCTGGCAGTGATCGTCCCTGCTCTGTTAGTAGCAGGTACTGCAAACGCTGCAGAAATCTATAACAAAGATGGCAACAAAGTAGATCTGTACGGTAAAGCTGTTGGTCTGCATTATTTTTCCAAGGGTAACGGTGAAAACAGTTACGGTGGCAATGGCGACATGACCTATGCCCGTCTTGGTTTTAAAGGGGAAACTCAAATCAATTCCGATCTGACCGGTTATGGTCAGTGGGAATATAACTTCCAGGGTAACAACTCTGAAGGCGCTGACGCTCAAACTGGTAACAAAACGCGTCTGGCATTCGCGGGTCTTAAATACGCTGACGTTGGTTCTTTCGATTACGGCCGTAACTACGGTGTGGTTTATGATGCACTGGGTTACACCGATATGCTGCCAGAATTTGGTGGTGATACTGCATACAGCGATGACTTCTTCGTTGGTCGTGTTGGCGGCGTTGCTACCTATCGTAACTCCAACTTCTTTGGTCTGGTTGATGGCCTGAACTTCGCTGTTCAGTACCTGGGTAAAAACGAGCGTGACACTGCACGCCGTTCTAACGGCGACGGTGTTGGCGGTTCTATCAGCTACGAATACGAAGGCTTTGGTATCGTTGGTGCTTATGGTGCAGCTGACCGTACCAACCTGCAAGAAGCTCAACCTCTTGGCAACGGTAAAAAAGCTGAACAGTGGGCTACTGGTCTGAAGTACGACGCGAACAACATCTACCTGGCAGCGAACTACGGTGAAACCCGTAACGCTACGCCGATCACTAATAAATTTACAAACACCAGCGGCTTCGCCAACAAAACGCAAGACGTTCTGTTAGTTGCGCAATACCAGTTCGATTTCGGTCTGCGTCCGTCCATCGCTTACACCAAATCTAAAGCGAAAGACGTAGAAGGTATCGGTGATGTTGATCTGGTGAACTACTTTGAAGTGGGCGCAACCTACTACTTCAACAAAAACATGTCCACCTATGTTGACTACATCATCAACCAGATCGATTCTGACAACAAACTGGGCGTAGGTTCAGACGACACCGTTGCTGTGGGTATCGTTTACCAGTTCTAATAGCACACCTCTTTGTTAAATGCCGAAAAAA

B1:

CTTAAATTTTACTTTTGGTTACATATTTTTCTTTTTGAAACCAAATCTTTATCTTTGTAGCACTTTCACGGTAGCGAAACGTTAGTTTGAATGGAAAGATGCCTGCAGACACATAAAGACACCAAACTCTCATCAATAGTTCCGTAAATTTTTATTGACAGAACTTATTGACGGCAGTGGCAGGTGTCATAAAAAAAACCATGAGGGTAATAAATAATGATGAAGCGCAATATTCTGGCAGTGATCGTCCCTGCTCTGTTAGTAGCAGGTACTGCAAACGCTGCAGAAATCTATAACAAAGATGGCAACAAAGTAGATCTGTACGGTAAAGCTGTTGGTCTGCATTATTTTTCCAAGGGTAACGGTGAAAACAGTTACGGTGGCAATGGCGACATGACCTATGCCCGTCTTGGTTTTAAAGGGGAAACTCAAATCAATTCCGATCTGACCGGTTATGGTCAGTGGGAATATAACTTCCAGGGTAACAACTCTGAAGGCGCTGACGCTCAAACTGGTAACAAAACGCGTCTGGCATTCGCGGGTCTTAAATACGCTGACGTTGGTTCTTTCGATTACGGCCGTAACTACGGTGTGGTTTATGATGCTCTGGGTTACACCGATATGCT

B2:

CTTTGGTATCGTTGGTGCTTATGGTGCAGCTGACCGTACCAACCTGCAAGAAGCTCAACCTCTTGGCAACGGTAAAAAAGCTGAACAGTGGGCTACTGGTCTGAAGTACGACGCGAACAACATCTACCTGGCAGCGAACTACGGTGAAACCCGTAACGCTACGCCGATCACTAATAAATTTACAAACACCAGCGGCTTCGCCAACAAAACGCAAGACGTTCTGTTAGTTGCGCAATACCAGTTCGATTTCGGTCTGCGTCCGTCCATCGCTTACACCAAATCTAAAGCGAAAGACGTAGAAGGTATCGGTGATGTTGATCTGGTGAACTACTTTGAAGTGGGCGCAACCTACTACTTCAACAAAAACATGTCCACCTATGTTGACTACATCATCAACCAGATCGATTCTGACAACAAAACCTCTACCGGTCCGTGCAAAACCTGCACCACCCCGGCTCTGGGCGTAGGTTCAGACGACACCGTTGCTGTGGGTATCGTTTACCAGTTCTAATAGCACACCTCTTTGTTAAATGCCGAAAAAACAGGACTTTGGTCCTGTTTTTTTTATACCTTCCAGAGCAATCTCACGTCTTGCAAAAACAGCCTGCGTTTTCATCAGTAATAGTTGGAATTTTGTAAATCTCCCGTTACCCTGATAGCGGACTTCCCTTCTGTAACCATAATGGAACCTCGTCATGTTTGAGAACATTACCGCCGCTCCTGCCGACCCGATTCTGGGCCTGGCCGATCTGTTTCGTGCCGATGAACGTCCCGGCAAAATTAACCTCGGGATTGGTGTCTATAAAGATGAGACGGGCAAAACCCCGGTACTGACCAGC

B3:

CTTTGGTATCGTTGGTGCTTATGGTGCAGCTGACCGTACCAACCTGCAAGAAGCTCAACCTCTTGGCAACGGTAAAAAAGCTGAACAGTGGGCTACTGGTCTGAAGTACGACGCGAACAACATCTACCTGGCAGCGAACTACGGTGAAACCCGTAACGCTACGCCGATCACTAATAAATTTACAAACACCAGCGGCTTCGCCAACAAAACGCAAGACGTTCTGTTAGTTGCGCAATACCAGTTCGATTTCGGTCTGCGTCCGTCCATCGCTTACACCAAATCTAAAGCGAAAGACGTAGAAGGTATCGGTGATGTTGATCTGGTGAACTACTTTGAAGTGGGCGCAACCTACTACTTCAACAAAAACATGTCCACCTATGTTGACTACATCATCAACCAGATCGATTCTGACAACAAACAACTTTATAAAACATGCAAACAGGCAGGTACATGTCCACCTGACATTATACTGGGCGTAGGTTCAGACGACACCGTTGCTGTGGGTATCGTTTACCAGTTCTAATAGCACACCTCTTTGTTAAATGCCGAAAAAACAGGACTTTGGTCCTGTTTTTTTTATACCTTCCAGAGCAATCTCACGTCTTGCAAAAACAGCCTGCGTTTTCATCAGTAATAGTTGGAATTTTGTAAATCTCCCGTTACCCTGATAGCGGACTTCCCTTCTGTAACCATAATGGAACCTCGTCATGTTTGAGAACATTACCGCCGCTCCTGCCGACCCGATTCTGGGCCTGGCCGATCTGTTTCGTGCCGATGAACGTCCCGGCAAAATTAACCTCGGGATTGGTGTCTATAAAGATGAGACGGGCAAAACCCCGGTACTGACCAGC

B4:

TTTAAGAAGGAGATATACATATGATGAAGCGCAATATTCTGGCAGTGATCGTCCCTGCTCTGTTAGTAGCAGGTACTGCAAACGCTGCAGAAATCTATAACAAAGATGGCAACAAAGTAGATCTGTACGGTAAAGCTGTTGGTCTGCATTATTTTTCCAAGGGTAACGGTGAAAACAGTTACGGTGGCAATGGCGACATGACCTATGCCCGTCTTGGTTTTAAAGGGGAAACTCAAATCAATTCCGATCTGACCGGTTATGGTCAGTGGGAATATAACTTCCAGGGTAACAACTCTGAAGGCGCTGACGCTCAAACTGGTAACAAAACGCGTCTGGCATTCGCGGGTCTTAAATACGCTGACGTTGGTTCTTTCGATTACGGCCGTAACTACGGTGTGGTTTATGATGCACTGGGTTACACCGATATGCTGCCAGAATTTGGTGGTGATACTGCATACAGCGATGACTTCTTCGTTGGTCGTGTTGGCGGCGTTGCTACCTATCGTAACTCCAACTTCTTTGGTCTGGTTGATGGCCTGAACTTCGCTGTTCAGTACCTGGGTAAAAACGAGCGTGACACTGCACGCCGTTCTAACGGCGACGGTGTTGGCGGTTCTATCAGCTACGAATACGAAGGCTTTGGTATCGTTGGTGCTTATGGTGCAGCTGACCGTACCAACCTGCAAGAAGCTCAACCTCTTGGCAACGGTAAAAAAGCTGAACAGTGGGCTACTGGTCTGAAGTACGACGCGAACAACATCTACCTGGCAGCGAACTACGGTGAAACCCGTAACGCTACGCCGATCACTAATAAATTTACAAACACCAGCGGCTTCGCCAACAAAACGCAAGACGTTCTGTTAGTTGCGCAATACCAGTTCGATTTCGGTCTGCGTCCGTCCATCGCTTACACCAAATCTAAAGCGAAAGACGTAGAAGGTATCGGTGATGTTGATCTGGTGAACTACTTTGAAGTGGGCGCAACCTACTACTTCAACAAAAACATGTCCACCTATGTTGACTACATCATCAACCAGATCGATTCTGACAACAAAACCTCTACCGGTCCGTGCAAAACCTGCACCACCCCGGCTCTGGGCGTAGGTTCAGACGACACCGTTGCTGTGGGTATCGTTTACCAGTTCTGAGATCCGGCTGCTAACAA

B5:

TTTAAGAAGGAGATATACATATGATGAAGCGCAATATTCTGGCAGTGATCGTCCCTGCTCTGTTAGTAGCAGGTACTGCAAACGCTGCAGAAATCTATAACAAAGATGGCAACAAAGTAGATCTGTACGGTAAAGCTGTTGGTCTGCATTATTTTTCCAAGGGTAACGGTGAAAACAGTTACGGTGGCAATGGCGACATGACCTATGCCCGTCTTGGTTTTAAAGGGGAAACTCAAATCAATTCCGATCTGACCGGTTATGGTCAGTGGGAATATAACTTCCAGGGTAACAACTCTGAAGGCGCTGACGCTCAAACTGGTAACAAAACGCGTCTGGCATTCGCGGGTCTTAAATACGCTGACGTTGGTTCTTTCGATTACGGCCGTAACTACGGTGTGGTTTATGATGCACTGGGTTACACCGATATGCTGCCAGAATTTGGTGGTGATACTGCATACAGCGATGACTTCTTCGTTGGTCGTGTTGGCGGCGTTGCTACCTATCGTAACTCCAACTTCTTTGGTCTGGTTGATGGCCTGAACTTCGCTGTTCAGTACCTGGGTAAAAACGAGCGTGACACTGCACGCCGTTCTAACGGCGACGGTGTTGGCGGTTCTATCAGCTACGAATACGAAGGCTTTGGTATCGTTGGTGCTTATGGTGCAGCTGACCGTACCAACCTGCAAGAAGCTCAACCTCTTGGCAACGGTAAAAAAGCTGAACAGTGGGCTACTGGTCTGAAGTACGACGCGAACAACATCTACCTGGCAGCGAACTACGGTGAAACCCGTAACGCTACGCCGATCACTAATAAATTTACAAACACCAGCGGCTTCGCCAACAAAACGCAAGACGTTCTGTTAGTTGCGCAATACCAGTTCGATTTCGGTCTGCGTCCGTCCATCGCTTACACCAAATCTAAAGCGAAAGACGTAGAAGGTATCGGTGATGTTGATCTGGTGAACTACTTTGAAGTGGGCGCAACCTACTACTTCAACAAAAACATGTCCACCTATGTTGACTACATCATCAACCAGATCGATTCTGACAACAAACAACTTTATAAAACATGCAAACAGGCAGGTACATGTCCACCTGACATTATACTGGGCGTAGGTTCAGACGACACCGTTGCTGTGGGTATCGTTTACCAGTTCTGAGATCCGGCTGCTAACAA

B6:

TTTAAGAAGGAGATATACATATGAAAGTTAAAGTACTGTCCCTC*CTGGTCCCAGCTCTGCTGGTAGCAGGCGCAGCAAACGCTGCTGAAGTTTACAACAAAGAC*GGCAACAAATTAGATCTGTACGGTAAAGTAGACGGCCTGCACTATTTCTCTGACAACAAAGATGTAGATGGCGACCAGACCTACATGCGTCTTGGCTTCAAAGGTGAAACTCAGGTTACTGACCAGCTGACCGGTTACGGCCAGTGGGAATATCAGATCCAGGGCAACAGCGCTGAAAACGAAAACAACTCCTGGACCCGTGTGGCATTCGCAGGTCTGAAATTCCAGGATGTGGGTTCTTTCGACTACGGTCGTAACTACGGCGTTGTTTATGACGTAACTTCCTGGACCGACGTACTGCCAGAATTCGGTGGTGACACCTACGGTTCTGACAACTTCATGCAGCAGCGTGGTAACGGCTTCGCGACCTACCGTAACACTGACTTCTTCGGTCTGGTTGACGGCCTGAACTTTGCTGTTCAGTACCAGGGTAAAAACGGCAACCCATCTGGTGAAGGCTTTACTAGTGGCGTAACTAACAACGGTCGTGACGCACTGCGTCAAAACGGCGACGGCGTCGGCGGTTCTATCACTTATGATTACGAAGGTTTCGGTATCGGTGGTGCGATCTCCAGCTCCAAACGTACTGATGCTCAGAACACCGCTGCTTACATCGGTAACGGCGACCGTGCTGAAACCTACACTGGTGGTCTGAAATACGACGCTAACAACATCTACCTGGCTGCTCAGTACACCCAGACCTACAACGCAACTCGCGTAGGTTCCCTGGGTTGGGCGAACAAAGCACAGAACTTCGAAGCTGTTGCTCAGTACCAGTTCGACTTCGGTCTGCGTCCGTCCCTGGCTTACCTGCAGACCTCTACCGGTCCGTGCAAAACCTGCACC

B7:

GGTCCGTGCAAAACCTGCACCACCCCGGCTTCTAAAGGTAAAAACCTGGGTCGTGGCTACGACGACGAAGATATCCTGAAATATGTTGATGTTGGTGCTACCTACTACTTCAACAAAAACATGTCCACCTACGTTGACTACAAAATCAACCTGCTGGACGACAACCAGTTCACTCGTGACGCTGGCATCAACACTGATAACATCGTAGCTCTGGGTCTGGTTTACCAGTTCTGAGATCCGGCTGCTAACAA

B8:

TTTAAGAAGGAGATATACATATGAAAGTTAAAGTACTGTCCCTCCTGGTCCCAGCTCTGCTGGTAGCAGGCGCAGCAAACGCTGCTGAAGTTTACAACAAAGACGGCAACAAATTAGATCTGTACGGTAAAGTAGACGGCCTGCACTATTTCTCTGACAACAAAGATGTAGATGGCGACCAGACCTACATGCGTCTTGGCTTCAAAGGTGAAACTCAGGTTACTGACCAGCTGACCGGTTACGGCCAGTGGGAATATCAGATCCAGGGCAACAGCGCTGAAAACGAAAACAACTCCTGGACCCGTGTGGCATTCGCAGGTCTGAAATTCCAGGATGTGGGTTCTTTCGACTACGGTCGTAACTACGGCGTTGTTTATGACGTAACTTCCTGGACCGACGTACTGCCAGAATTCGGTGGTGACACCTACGGTTCTGACAACTTCATGCAGCAGCGTGGTAACGGCTTCGCGACCTACCGTAACACTGACTTCTTCGGTCTGGTTGACGGCCTGAACTTTGCTGTTCAGTACCAGGGTAAAAACGGCAACCCATCTGGTGAAGGCTTTACTAGTGGCGTAACTAACAACGGTCGTGACGCACTGCGTCAAAACGGCGACGGCGTCGGCGGTTCTATCACTTATGATTACGAAGGTTTCGGTATCGGTGGTGCGATCTCCAGCTCCAAACGTACTGATGCTCAGAACACCGCTGCTTACATCGGTAACGGCGACCGTGCTGAAACCTACACTGGTGGTCTGAAATACGACGCTAACAACATCTACCTGGCTGCTCAGTACACCCAGACCTACAACGCAACTCGCGTAGGTTCCCTGGGTTGGGCGAACAAAGCACAGAACTTCGAAGCTGTTGCTCAGTACCAGTTCGACTTCGGTCTGCGTCCGTCCCTGGCTTACCTGCAGCAACTTTATAAAACATGCAAACAGGCAGGTACATGTCCACCT

B9:

AAAACATGCAAACAGGCAGGTACATGTCCACCTGACATTATATCTAAAGGTAAAAACCTGGGTCGTGGCTACGACGACGAAGATATCCTGAAATATGTTGATGTTGGTGCTACCTACTACTTCAACAAAAACATGTCCACCTACGTTGACTACAAAATCAACCTGCTGGACGACAACCAGTTCACTCGTGACGCTGGCATCAACACTGATAACATCGTAGCTCTGGGTCTGGTTTACCAGTTCTGAGATCCGGCTGCTAACAA
